# Low Circulating Concentrations of Very Long Chain Saturated Fatty Acids Are Associated with High Risk of Mortality in Kidney Transplant Recipients

**DOI:** 10.3390/nu13103383

**Published:** 2021-09-26

**Authors:** Fabian A. Vogelpohl, António W. Gomes-Neto, Ingrid A. Martini, Camilo G. Sotomayor, Dion Groothof, Maryse C. J. Osté, Margaretha Rebecca Heiner-Fokkema, Frits A. J. Muskiet, Stefan P. Berger, Gerjan Navis, Ido P. Kema, Stephan J. L. Bakker

**Affiliations:** 1 Department of Internal Medicine, University Medical Center Groningen, University of Groningen, 9713 GZ Groningen, The Netherlands; a.w.gomes.neto@umcg.nl (A.W.G.-N.); c.g.sotomayor.campos@umcg.nl (C.G.S.); d.groothof@umcg.nl (D.G.); m.c.j.oste@umcg.nl (M.C.J.O.); s.p.berger@umcg.nl (S.P.B.); g.j.navis@umcg.nl (G.N.); s.j.l.bakker@umcg.nl (S.J.L.B.); 2 Department of Laboratory Medicine, University Medical Center Groningen, University of Groningen, 9713 GZ Groningen, The Netherlands; i.a.martini@umcg.nl (I.A.M.); m.r.heiner@umcg.nl (M.R.H.-F.); f.a.j.muskiet@umcg.nl (F.A.J.M.); i.p.kema@umcg.nl (I.P.K.)

**Keywords:** arachidic acid, behenic acid, epidemiology, gas chromatography, infection, kidney transplantation, lignoceric acid, sphingolipids, very long chain saturated fatty acids

## Abstract

Kidney transplant recipients (KTR) are at increased risk of mortality, particularly from infectious diseases, due to lifelong immunosuppression. Although very long chain saturated fatty acids (VLSFA) have been identified as crucial for phagocytosis and clearance of infections, their association with mortality in immunocompromised patient groups has not been studied. In this prospective cohort study we included 680 outpatient KTR with a functional graft ≥1 year and 193 healthy controls. Plasma VLSFA (arachidonic acid (C20:0), behenic acid (C22:0) and lignoceric acid (C24:0)) were measured by gas chromatography coupled with a flame ionization detector. Cox regression analyses was used to prospectively study the associations of VLSFA with all-cause and cause-specific mortality. All studied VLSFA were significantly lower in KTR compared to healthy controls (all *p* < 0.001). During a median (interquartile range) follow-up of 5.6 (5.2–6.3) years, 146 (21%) KTR died, of which 41 (28%) died due to infectious diseases. In KTR, C22:0 was inversely associated with risk of all-cause mortality, with a HR (95% CI) per 1-SD-increment of 0.79 (0.64–0.99), independent of adjustment for potential confounders. All studied VLSFA were particularly strongly associated with mortality from infectious causes, with respective HRs for C20:0, C22:0 and C24:0 of 0.53 (0.35–0.82), 0.48 (0.30–0.75), and 0.51 (0.33–0.80), independent of potential confounders. VLSFA are inversely associated with infectious disease mortality in KTR after adjustment, including HDL-cholesterol. Further studies are needed to assess the effect of VLSFA-containing foods on the risk of infectious diseases in immunocompromised patient groups.

## 1. Introduction

Kidney transplantation can be regarded as the “gold standard” therapy for patients with end-stage kidney disease. Compared to chronic dialyses, kidney transplantation offers several benefits, such as superior survival, cost-effectiveness and improved quality of life [1,2,3]. However, the survival of kidney transplant recipients (KTR) is still significantly lower compared to age-matched controls in the general population, with infections being one of the major causes of this excessive mortality [4]. The lifelong immunosuppression KTR receive to prevent rejection is an important contributor to the increased susceptibility to fatal infections. Even after a significant time period post transplantation, solid organ transplant recipients are at persistent risk of adverse long-term outcomes and increased frequency of infections caused by community acquired pathogens [5,6]. It is established that this increased susceptibility is partly due to inhibition of the adaptive immune system by immunosuppressive medication [7,8]. However, it has also been shown that immunosuppression in KTR decreases the innate immune function, especially the phagocytotic capacity of the innate immune system, which could otherwise compensate the inhibition of adaptive immunity [9,10]. Therefore, identifying modifiable factors, such as diet and lifestyle, which can strengthen the immune system and potentially improve patient survival after kidney transplantation is of particular importance.

Recently, it has been shown that very long chain saturated fatty acids (VLSFA), which are predominantly incorporated into the sphingolipids ceramide and sphingomyelin, are essential for maturation of macrophages and invariant natural killer T cells, for efficient phagocytosis by macrophages and for neutrophil migration [11,12,13]. This suggests that VLSFA may play an important role in clearance of pathogens by the innate immune system.

Importantly, several studies have found strong inverse associations of plasma VLSFA behenic acid (C22:0) and lignoceric acid (C24:0) with body mass index (BMI), plasma triglycerides, fasting glucose, glycated hemoglobin (HbA_1C_) levels, and prevalence of diabetes [14,15,16]. Interestingly, high BMI, high plasma triglycerides, high fasting glucose, high HbA_1C_ and diabetes are also associated with increased hepatic de novo lipogenesis (DNL), making it possible that these observed associations are a consequence of dilution of VLSFA by higher synthesis of fatty acids involved in hepatic DNL [17]. VLSFA are synthesized endogenously by elongation of stearic acid [18]. However, VLSFA are also present in certain foods-including peanuts, macadamia nuts, cashew nuts, canola oil and in trace amounts in other nuts and oils [19]-and it has been shown that circulating levels of VLSFA can be increased through short-term feeding trials of peanuts and macadamia nuts [20,21].

We hypothesized that (1) because of the high prevalence of metabolic syndrome in KTR [22,23], circulating VLSFA would be relatively low in KTR compared to healthy controls; (2) circulating VLSFA in KTR would be related to peanut intake; and (3) circulating VLSFA would be prospectively associated with risk of all-cause mortality and risk of mortality from infectious causes in outpatient KTR. Therefore, in this study we measured plasma VLSFA levels in a prospective cohort of stable KTR and healthy controls. We aimed to evaluate whether circulating VLSFA differ in KTR compared to healthy controls and if VLSFA are associated with peanut intake and all-cause mortality in KTR with specific regard to infectious disease mortality.

## 2. Materials and Methods

### 2.1. Study Design and Subjects

For this study we used data from the Transplant Lines Food and Nutrition Biobank and Cohort Study (NCT02811835), an observational single center cohort study of adult stable KTR with a functioning graft ≥1 year who were recruited at the outpatient clinic of the University Medical Center of Groningen (the Netherlands) between 2008 and 2011. For the participant flowchart, see Supplemental Figure S1. Patients with overt congestive heart failure or who were diagnosed with cancer, other than skin cancer, were considered ineligible to participate. Of the 817 initially invited, KTR, 707 (87%) provided informed consent to participate. For the present study we excluded subjects with missing EDTA plasma samples or data, resulting in 680 KTR eligible for analyses. We also included 193 healthy kidney donors as a control group reflecting the general population, none of which had to be excluded because of missing EDTA plasma samples or data. The study was conducted according to the guidelines settled in the Declaration of Helsinki and the Declaration of Istanbul on Organ Trafficking and Transplant Tourism. The Institutional Review Board of the UMCG approved the study protocol (METc 2008/186).

KTR were all transplanted at the University Medical Center Groningen and had no history of drug or alcohol addiction according to their patient files. KTR were on standard antihypertensive and immunosuppressive therapy. Immunosuppressive therapy consisted of the following: azathioprine (100 mg/day) and prednisolone (starting with 20 mg/day and tapering to 10 mg/day) from 1968 to 1989; cyclosporine (target trough levels 175–200 mg/L in the first 3 months, 100 mg/L thereafter) and prednisolone (starting with 20 mg/day and tapering to 10 mg/day) from 1989 to 1996. In 1997 mycophenolate mofetil (2 g/day) was added to the standard immunosuppressive regimen, and for KTR with no complications, cyclosporine was slowly withdrawn from one year after transplantation onward. In 2012, cyclosporine was replaced by tacrolimus, and KTR continued triple-immunosuppressive therapy with prednisolone (20 mg/day, tapering to 5 mg/day), tacrolimus (target trough levels 8–12 mg/L in the first three months, 6–10 mg/L until month six, and 4–6 mg/L from six months onward), and mycophenolate mofetil (starting with 2 g/day, tapering to 1 g/day) [24]. Except for discouraging excess sodium intake and encouraging losing weight in overweight individuals, no specific dietary counselling was included as a routine regimen. Relevant characteristics including recipient age, gender, and transplant information were extracted from patient records. Self-report questionnaires were used to obtain information on smoking behaviour and alcohol intake. Physical activity was assessed by using the Short Questionnaire to Assess Health-enhancing Physical Activity (SQUASH) score (time × intensity).

### 2.2. Assessment of Dietary Intake

Information on dietary intake was assessed using a semi-quantitative food-frequency questionnaire that inquired about intake of 177 food items during the last month, including separate questions for the consumption of ‘peanuts’, for the consumption of ‘peanut butter’ and for the consumption of ‘other nuts, mixed nuts’ (tree nuts) [25]. The latter category includes almonds, Brazil nuts, cashews, hazelnuts, macadamia nuts, pecans, pistachios, and walnuts. Dietary data were converted into energy and nutrient intake by research dieticians and nutritionists using the Dutch Food Composition Table [26].

### 2.3. Clinical Parameters

All measurements were performed once at baseline during a morning visit to the outpatient clinic. BMI was calculated as weight (kilograms) divided by height (meters squared). Blood pressure and heart rate were measured by using a semiautomatic device (Dinamap 1846; Critikon, Tampa, FL, USA).

Blood was drawn after a fasting period of 8–12 h, which included no medication intake. Diabetes was defined as use of antidiabetic medication, fasting plasma glucose ≥7.0 mmol/L [27] or HbA_1C_ higher than 6.5, as proposed by Shabir et al. [28]. Metabolic syndrome was defined according to the definition from the National Cholesterol Education Program Adult Treatment Panel III (NCEP-ATP-III) [29]. According to those criteria, an individual has metabolic syndrome if he or she suffers from three or more of the following criteria: (1) a waist circumference > 102 cm in men and > 88 cm in women; (2) serum triglycerides ≥ 1.70 mmol/L or use of lipid-lowering medication; (3) serum HDL-cholesterol < 1.03 mmol/L in men and < 1.29 mmol/L in women; (4) blood pressure ≥ 130/85 mmHg or use of antihypertensive medication; and (5) fasting plasma glucose ≥ 5.6 mmol/L or use of antidiabetic medication (including insulin). To estimate hepatic steatosis, the algorithm of the Fatty Liver Index (FLI) was used. The FLI was calculated as follows: FLI = (e ^0.953*loge (triglycerides) + 0.139*BMI + 0.718*loge (ggt) + 0.053*waist circumference − 15.745^)/(1 + e ^0.953*loge (triglycerides) + 0.139*BMI + 0.718*loge (ggt) + 0.053*waist circumference − 15.745^) * 100 [30]. Serum creatinine was measured by using an enzymatic isotope dilution assay, traceable on mass spectrometry, on a Roche P-Modulator automated analyzer (Roche Diagnostics, Basel, Switzerland), and kidney function was assessed by using the Chronic Kidney Disease Epidemiology Collaboration (CKD-EPI) formula for creatinine in order to calculate the estimated glomerular filtration rate (eGFR) [31]. HDL-cholesterol was derived from lipoprotein measures assessed on a Vantera Clinical NMR Analyzer platform [32]. Other serum and urine variables were assessed using standard laboratory methods. To assure adequate 24-h urine samples, all subjects were instructed to discard the morning urine specimen, collect all subsequent urine through the next 24 h and include the next morning’s urine specimen. Total urinary protein concentration was determined using the Biuret reaction (MEGA AU 150, Merck Diagnostica, Darmstadt, Germany). Proteinuria was defined as urinary protein excretion ≥ 0.5 g/24 h.

### 2.4. Assessment of Plasma VLSFA

EDTA-plasma samples were stored frozen at −80 °C until use for assessment of fatty acid profiles. Analyses of fatty acids were performed in the Department of Laboratory Medicine of the University Medical Center Groningen using the methodology as described by Ichihara and Fukubayashi [33]. In short, total lipids were extracted by the method of Folch et al., using 2 mL of chloroform-methanol (2:1) and a 100 μL EDTA-plasma sample [34]. Fatty acids were transmethylated with methanolic-HCL into fatty acid methyl esters (FAME) by incubation of the solution at 45 °C for 4 h. The samples were extracted with hexane and eventually re-dissolved into 100 μL hexane. An internal standard for the quantification of fatty acids (100 μL of a solution of 50.0 mg free fatty acid 19:0/100 mL methanol), obtained from Larodan (Solna, Sweden), was added after isolation of FAME. 100 μL butylated hydroxytoluene (1 g/100 mL methanol) from Sigma-Aldrich (Zwijndrecht, The Netherlands) were added to prevent fatty acid oxidation. Aliquots of 2 μL were injected into an Agilent model 6890 gas chromatography and detected with an Agilent 7683 series 116 flame ionization detector. FAME were identified by comparing retention times with those of known standards (Supelco 37 component FAME mix (Sigma-Aldrich)). Three VLSFA were measured using this method: arachidic acid (C20:0), C22:0, and C24:0. Concentrations of circulating fatty acids were expressed as mol% of total circulating fatty acids.

### 2.5. Study Endpoints

The primary endpoint of this study was all-cause mortality. The secondary endpoint was cause-specific mortality. Therefore, we investigated infectious disease mortality, cancer mortality and cardiovascular disease (CVD) mortality. Information on the cause of death was derived from the patients’ medical records and was assessed by a nephrologist. Infectious disease mortality was identified according to the Ninth Revision of the International Classification of Diseases (ICD-9) codes 1–139 [35], which includes mortality due to viral infections, bacterial infections, parasitic infections and fungal infections. Cancer mortality was defined according ICD-9 codes 140–239. CVD mortality was defined as death due to cerebrovascular disease, ischemic heart disease, heart failure, or sudden cardiac death according to ICD-9 codes 410–447. The continuous surveillance system of the outpatient program ensured up-to-date information on patient status. Endpoints were recorded until September 2015 by a qualified physician. Because endpoints were recorded until September 2015, mortality due to viral infections does not include death due to Coronavirus Disease 2019 (COVID-19) infections. No participants were lost to follow-up.

### 2.6. Statistical Analyses

Normally distributed data are presented as means ± SD, whereas skewed data are presented as median (IQR) and percentages are used to summarize categorical data. *p*-values for differences between KTR and living kidney controls before donation were tested by *t*-test and the Mann–Whitney test for normally and skewed distributed continuous variables, respectively. Fisher’s exact test was used to test for differences in binary variables.

Linear regression analyses were performed to evaluate the association of circulating fatty acids with baseline characteristics. Natural log transformation was used for analyses of variables with a skewed distribution. To prospectively analyze the association between VLSFA levels and study endpoints, we performed Cox proportional hazards regression analyses in which we analyzed the association of VLSFA levels with study endpoints. For illustration purposes and to enable more objective comparisons, plasma VLSFA concentrations were standardized to z-scores and analyzed as such. In the longitudinal analyses, plasma VLSFA were entered as a continuous variable. Cox regression models were built in a stepwise fashion to avoid overfitting and to keep the number of predictors in proportion to the number of events [36]. Proportionality of hazards for covariates was investigated by inspecting the Schoenfeld residuals.

We performed multivariable Cox regression analyses in which we cumulatively adjusted for common potential confounders, including age, sex (model 1); primary kidney disease, time between transplantation and study baseline, eGFR and urinary protein excretion (model 2); albumin concentration and procalcitonin concentration (model 3); and smoking status and alcohol intake (model 4). Further models were constructed additive to model 4. We performed additional adjustment for triglyceride levels (model 5); use of cyclosporine (model 6); and systolic blood pressure, HDL-cholesterol, BMI and diabetes status (model 7). Hazard ratios (HR) of Cox regression models are reported with a 95% confidence interval (CI).

In secondary analyses, we tested for potential effect modification by age, sex, BMI, diabetes, renal function, smoking status, alcohol intake, use of immunosuppressive medication and physical activity.

Data were analyzed with SPSS version 23.0 (IBM Corp., Armonk, NY, USA) and R version 3.2.3 (R Foundation for Statistical Computing, Vienna, Austria). In all analyses a *p*-value < 0.05 was considered to indicate statistical significance.

## 3. Results

### 3.1. VLSFA in KTR and Healthy Controls

The total cohort consisted of 680 stable outpatient KTR (57% male, median age 54.7 (44.6–63.0) years), included at a median time of 5.4 (1.9–12.0) years after transplantation, and 193 healthy controls (43% male, median age 54.0 (45.6–63.1) years) (Table 1). Plasma levels of all three VLSFA were lower in KTR than in healthy controls (*p* < 0.001 for all). For C20:0, the mean difference was 8.7% (calculated from the median values of 0.23 mol% and 0.25 mol% in KTR and healthy controls, respectively, with healthy controls as reference). For C22:0, the mean difference was 10.9% (calculated from the median values of 0.57 mol% and 0.64 mol% in KTR and healthy controls, respectively). For C24:0, the mean difference was 12.7% (calculated from the median values of 0.48 mol% and 0.55 mol% in KTR and healthy controls, respectively). Compared to healthy controls, consumption of peanuts, peanut butter and tree nuts was lower in KTR, although the differences did not reach statistical significance.

### 3.2. Associations between VLSFA and Clinical Baseline Characteristics in KTR

Associations of circulating VLSFA with clinical baseline characteristics in KTR are shown in Table 2. VLSFA showed strong positive associations with each other, especially between C22:0 and C24:0 (Std. β = 0.94, *p* < 0.001). KTR with low levels of C22:0 and C24:0 were older and BMI was inversely associated with all studied VLSFA, with effect sizes increasing from C20:0 towards C24:0. While neither diastolic blood pressure, systolic blood pressure nor heart rate were consistently associated with VLSFA, use of anti-hypertensive medication was inversely associated with each VLSFA. Among immunosuppressant medication, use of cyclosporine was inversely associated with all VLSFA. Creatinine as well as cystatin C showed inverse associations with each VLSFA, while only C20:0 and C22:0 were positively associated with eGFR. All VLSFA were inversely associated with fasting glucose levels, HbA1C, diabetes and use of antidiabetic medication, with increasing effect size from C20:0 towards C24:0. C20:0 and C24:0 were positively associated with albumin, while each VLSFA was inversely associated with procalcitonin. C22:0 and C24:0 were positively associated with LDL cholesterol, while each VLSFA were strongly associated with HDL cholesterol. Triglycerides were strongly inversely associated with each VLSFA. Being a smoker was inversely associated with C20:0 and C22:0. Each VLSFA was inversely associated with the presence of metabolic syndrome and with the fatty liver index. Lastly, C22:0 and C24:0 concentrations were associated with intake of peanuts and peanut butter, while all VLSFA were associated with tree nut intake.

### 3.3. Prospective Analyses of All-Cause and Cause-Specific Mortality

**Figure 1:**
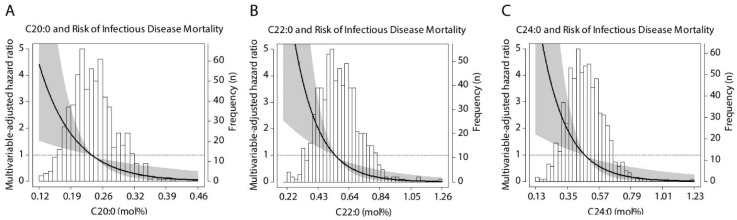
Association of standardized circulating VLSFA with risk of infectious disease mortality. Data were fitted by Cox proportional-hazards regression using mean C20:0 (0.24 mol%, **A**), C22:0 (0.57 mol%, **B**) and C24:0 (0.48 mol%, **C**) concentrations as reference value. The black line represents the hazard ratio and the grey area represents the 95% confidence interval.

During follow-up of 5.6 (5.2–6.3) years, 146 (21%) KTR died with 41 (28%) deaths due to severe infections, 25 (17%) deaths due to cancer and 59 (40%) deaths due to CVD mortality. Higher plasma concentrations of all VLSFA were associated with lower risk of all-cause mortality, independent of potential confounders including age, sex, eGFR, proteinuria, time between transplantation and baseline, procalcitonin concentration and albumin concentration, and smoking status and alcohol intake (Table 3, model 4). This inverse association did not materially change after adjustment for plasma triglycerides or use of cyclosporine (models 5 and 6). Adjustment for CVD risk factors, including systolic blood pressure, HDL-cholesterol, BMI and diabetes status caused the association between C20:0 and all-cause mortality to become insignificant, while the association between C24:0 and all-cause mortality became borderline significant. Analyses of cause-specific mortality revealed a strong association between all VLSFA with mortality from infectious diseases, independent of potential confounders (Table 4). Comparing the HRs of all four fatty acids, C22:0 consistently showed the lowest HR across all models (e.g., HR (95% CI) per 1-SD relative increment of 0.51 (0.35–0.73) in model 3, *p* < 0.001, Figure 1). None of the investigated VLSFA was associated with CVD mortality or cancer mortality after adjustment for potential confounders (Supplemental Tables S1 and S2).

In secondary analyses, we did not consistently identify effect modification by either age, sex, BMI, diabetes, eGFR, smoking status, alcohol intake, prednisolone dosage or physical activity on the association between levels and VLSFA with all-cause mortality, neither with infectious disease mortality.

## 4. Discussion

In this study, we showed that circulating levels of the VLSFA C20:0, C22:0 and C24:0 are lower in a large cohort of stable outpatient KTR compared to healthy controls. Further, peanut intake, peanut butter intake and tree nut intake are positively associated with C22:0 and C24:0 (in KTR as well as in healthy controls). All VLSFA are inversely associated with risk of all-cause mortality. Additionally, we show even stronger associations with infectious disease mortality, independent of adjustment for demographic factors, lifestyle factors and clinical conditions.

We observed that VLSFA are lower in KTR compared to healthy controls. We further found, aligned with other studies, strong inverse associations between C22:0 and C24:0 with BMI, plasma triglycerides, fasting glucose, Hb1Ac levels, diabetes, the presence of metabolic syndrome, and the fatty liver index (Table 2), indicating that low levels of VLSFA may be associated with metabolic dysfunction [14,15,16]. All VLSFA were associated with use of cyclosporine, which is known to cause increases in triglycerides [37] which consist of fatty acids that might dilute levels of VLSFA. As VLSFA are strongly associated with triglycerides in our study, we hypothesize that increased triglyceride levels due to the use of cyclosporine also result in a dilution of VLSFA accompanying increases in other fatty acids. VLSFA, especially C22:0 and C24:0, are mainly incorporated into the sphingolipid species ceramide, which can be further converted to sphingomyelin [38]. In mammals, fatty acids are elongated in the endoplasmic reticulum by various elongases, with C22:0 and C24:0 being mainly produced by elongation of very long chain fatty acids protein 1 (elovl1) and incorporated into ceramides by ceramide synthase 2 (CerS2) [18,39]. In accordance with the associations of circulating C22:0 and C24:0 with parameters of metabolic dysfunction observed in human studies, mice fed on a high-fat diet exhibit decreased hepatic expression of *CerS2* and decreased levels of ceramides containing C22:0 and C24:0 while *CerS2*-null mice develop insulin resistance and impaired hepatic insulin signaling [40,41]. These findings together exemplify the importance of these ceramides in the pathophysiology of metabolic disorders. In addition to lower concentrations of circulating VLSFA in KTR compared to healthy controls observed here, Szczuko et al. recently reported lower circulating C20:0, C22:0 and C24:0 in women with chronic kidney disease and women with metabolic syndrome compared to healthy controls [42]. As diabetes and the metabolic syndrome are highly prevalent in KTR compared to healthy controls (Table 1) [22,23] we suggest that the lower circulating VLSFA observed in KTR compared to healthy controls can at least partly be explained by increasing metabolic dysfunction prevalent in KTR.

We found here that relatively high levels of circulating C20:0, C22:0 and C24:0 are associated with lower risk of all-cause mortality, particularly mortality due to infectious diseases. Sphingolipids are involved in various cellular processes associated with immunity, inflammation and inflammatory disorders, and it is established that sphingolipid species have varying biological functions depending on the type of fatty acid incorporated [13,43]. Recent in vitro studies showed that synthesis of VLSFA by elovl1 and incorporation of VLSFA into ceramides by ceramide synthase 2 are necessary for human macrophage maturation and efficient phagocytosis [11,12]. Further, inhibition of ceramide synthase 2 is decreasing the migratory capacity of neutrophils. In line with these findings, *CerS2*-deficient mice show a higher susceptibility to LPS- mediated septic shock [44]. The importance of ceramides containing VLSFA, especially C22:0 and C24:0, for clearance of pulmonary infections has been shown previously in *CerS2*-deficient mice and in patients with cystic fibrosis, and modulation of this imbalance might be a possible drug target for managing pulmonary infections [45,46]. As ceramide synthase 2 shows the highest activity towards incorporation of C22:0 and C24:0 into ceramides [39], these findings are in concordance with our observation that levels of C22:0 and C24:0 show the strongest inverse association with all-cause and infectious disease mortality. Therefore, we hypothesize that low circulating VLSFA might predispose KTR, which are particularly susceptible to infection due to long-term immunosuppression, to severe infections as a result of a decreased ability of innate immune cells to form ceramides with incorporated VLSFA. However, as all VLSFA studied here show strong correlation with each other, further studies are needed to determine potential functional difference between these VLSFA.

It has been reported that VLSFA are associated with all-cause and cause-specific mortality in adults of the general population aged ≥65 years [47]. In this cohort, both C22:0 and C24:0 were found to be significantly associated with all-cause, CVD and non-CVD mortality. In analyses in which non-CVD mortality was divided in further subtypes of mortality, C22:0 and C24:0 were nominally significantly associated with infectious disease mortality, albeit that point estimates of hazard ratios for both C22:0 and C24:0 were lower for infectious disease mortality than for CVD mortality. However, in this cohort the rate of infectious deaths was much lower (8%) than the rate of CVD deaths (39%), while these respective rates were 28% and 40% in our cohort, pointing to the much higher susceptibility for a severe course of infection in immunocompromised transplantation patients. The low rate of infectious disease mortality events in the analyses of the general population cohort might have been responsible for very wide 95% confidence intervals for the hazard ratio, rendering the association insignificant despite a lower point estimate. VLSFAs are derived from food as well as produced endogenously. Foods containing VLSFA include peanuts, macadamia nuts, and canola oil [18]. In our study, intake of peanuts and peanut butter were associated with concentrations of C22:0 and C24:0 in KTR. Although it is not exactly known to which extent dietary intake and endogenous metabolism influence levels of circulating VLSFA, short-term feeding trials with peanuts and macadamia nuts showed that levels of VLSFA can be modified by dietary intake [20,21]. Interestingly, it has been shown that intake of peanuts is associated with a decreased risk of respiratory disease in the general population [48]. However, as comparable associations were reported for tree nuts as well, it is currently unknown if these effects stem from VLSFA or from components common in both peanuts and tree nuts such as mono- and polyunsaturated fatty acids. Intriguingly, it has been shown that macrophage membrane fatty acid composition can be influenced in a dose-dependent manner in humans by dietary fatty acid exposure [49]. More studies are needed to study the possibility of increasing circulating VLSFA by dietary measures in order to improve the susceptibility of KTR to life-threatening infections.

Our study has several strengths, including a median follow-up of 5.6 years with all-cause and infectious disease mortality as clinically relevant endpoints and without loss of participants due to follow-up. Furthermore, our study included a large sample size of the specific setting of stable outpatient KTR. Moreover, data was extensively collected, which allowed adjustment for many potential confounders of the main results. We also acknowledge limitations of the current study as follows. Plasma VLSFA were only measured at baseline and therefore changes in dietary intake during the follow-up period might not be accounted for in our analysis. Although we adjusted for demographic, lifestyle related, and clinical factors that may influence plasma VLSFA and total or infectious disease mortality, we cannot exclude residual confounding. Because the investigated VLSFA strongly correlated with each other, interpretation of the independent associations of each individual VLSFA with total and infectious mortality is challenging. As we measured the total plasma fatty acid content, we also cannot deduce the plasma compartment as well as the lipid class in which VLSFA showed the observed associations, although the literature suggests a major incorporation of VLSFA into sphingolipids [18]. Furthermore, as total plasma fatty acids have a lower half-life compared to erythrocyte fatty acids, measuring VLSFA in erythrocytes would have given a better estimate of long-term dietary FA intake. It should also be acknowledged that we assessed nut intake using self-reported FFQ, which might lead to recall bias and potential possible over- or under-reporting of dietary intake. Another limitation of the self-reported FFQ is that it did not allow for splitting up the category of tree nuts to specific types of tree nuts. As the cause of infectious mortality was not recorded in this study, we also cannot say if association of VLSFA with infectious disease mortality is specific for certain infectious diseases, such as pneumonia, or holds true for varying infections. Additionally, our study population consisted predominantly of Caucasian participants, which calls for prudence to extrapolate our results to different populations with regard to ethnicity. Finally, since the current study is observational by design, no conclusions of causality can be drawn from our results.

## 5. Conclusions

In conclusion, our study shows for the first time that circulating VLSFA are lower in KTR compared to healthy controls and that circulating VLSFA are inversely associated with infectious disease mortality. These associations were independent of adjustment for potential confounders including demographics, lifestyle factors, clinical conditions and HDL cholesterol. Although our results suggest a strong link between circulating VLSFA levels and mortality due to infectious disease, future studies have to elucidate the responsible underlying biological mechanism. Further, it should be investigated whether our reported associations can be observed in patients with other kidney diseases or in other immunocompromised patients. Finally, it has to be studied if interventions such as intake of VSLFA-containing foods, i.e., peanuts and peanut butter, could be applicable for the prevention of life-threatening infections in KTR.

## Figures and Tables

**Table 1:** Baseline characteristics of KTR and healthy controls.

Baseline Characteristics	KTR *n* = 680	Healthy Control *n* = 193	*p*
Demographics			
Age, years	54.7 (44.6–63.0)	54.0 (45.6–63.1)	0.76
Male gender, n (%)	401 (56.8)	111 (57.5)	0.87
Body mass index, kg/m^2^	25.9 (23.2–29.4)	25.3 (23.5–27.8)	0.10
Waist circumference, cm	98 ± 15	91 ± 10	<0.001
Metabolic syndrome (yes), n (%)	436 (64.1)	42 (21.8)	<0.001
Fatty liver index, arbitrary units	56 (31–80)	32 (17–58)	<0.001
Circulating VLSFA			
C20:0, mol%	0.24 ± 0.05	0.25 ± 0.05	<0.001
C22:0, mol%	0.57 ± 0.14	0.64 ± 0.14	<0.001
C24:0, mol%	0.48 ± 0.13	0.55 ± 0.13	<0.001
Renal function parameters			
Creatinine, umol/L	156 (112–181)	82 (72–93)	<0.001
eGFR, mL/min/1.73 m^2^	41.0 ± 18.6	93.1 ± 16.1	<0.001
Proteinuria 0.5 g/day, n (%)	152 (22.4)	1 (0.5)	<0.001
Glucose homeostasis			
Glucose, mmol/L	5.3 (4.9–6.0)	5.3 (5.0–5.7)	0.58
HbA1C, %	5.8 (5.5–6.2)	5.6 (5.4–5.8)	<0.001
Diabetes, n (%)	162 (23.8)	11 (5.7)	<0.001
Lipids			
Total cholesterol, mmol/L	5.1 ± 1.1	5.3 ± 1.1	0.04
Triglycerides, mmol/L	1.7 (1.3–2.3)	1.2 (0.8–1.6)	<0.001
HDL cholesterol, mmol/L	1.3 (1.1–1.6)	1.4 (1.2–1.7)	<0.001
Statin use, n (%)	359 (52.8)	7 (3.6)	<0.001
Liver parameter			
Gamma GT, U/L	21 (15–33)	26 (19–41)	<0.001
Health lifestyle			
Current smoker, n (%)	81 (11.9)	39 (20.2)	0.004
Total energy intake, kJ/day	8713 (7172–10607)	9055 (7458–10635)	0.38
Peanuts, g/day	0.6 (0.0–3.4)	1.3 (0.0–4.8)	0.09
Peanut butter, g/day	0.0 (0.0–4.0)	0.0 (0.0–4.3)	0.77
Tree nuts, g/day	0.0 (0.0–3.9)	0.8 (0.0–3.5)	0.35

Data are presented as mean ± SD, median (25th–75th percentile) or number (%). Differences between KTR and healthy controls were tested by independent *t*-test, Mann-Whitney U test or Chi-square test. Abbreviations: C20:0, arachidic acid; C22:0, behenic acid; C24:0, lignoceric acid; eGFR, estimated glomerular filtration rate; HDL, high-density lipoprotein; kJ, kilojoule; KTR, kidney transplant recipients; VLSFA, very long chain saturated fatty acids.

**Table 2:** Baseline characteristics of the KTR cohort and univariable association of circulating VLSFA concentrations across clinical parameters.

Baseline Characteristics	Total Population	C20:0 (mol%)	C22:0 (mol%)	C24:0 (mol%)
		Std. β	Std. β	Std. β
Demographics				
Age, years	54.7 (44.6–63.0)	0.02	−0.11 **	−0.09 *
Male gender, *n* (%)	401 (56.8)	0.13 ***	0.04	−0.03
BMI, kg/m^2^	25.9 (23.2–29.4)	−0.10 *	−0.12 **	−0.17 ***
Waist circumference, cm	98 ± 15	−0.20 ***	−0.19 ***	−0.21 ***
Caucasian, *n* (%)	677 (99.6)	0.04	−0.01	−0.02
Circulating VLSFA				
C20:0, mol%	0.24 ± 0.05	–	0.74 ***	0.63 ***
C22:0, mol%	0.57 ± 0.14	0.74 ***	–	0.94 ***
C24:0, mol%	0.48 ± 0.13	0.63 ***	0.94 ***	–
Primary kidney disease				
Glomerulonephritis, *n* (%)	175 (25.7)	−0.01	−0.01	0.01
Interstitial nephritis, *n* (%)	87 (12.8)	0.05	0.06	0.03
Cystic kidney disease, *n* (%)	139 (20.4)	−0.02	−0.02	−0.03
Other congenital and hereditary kidney disease, *n* (%)	37 (5.4)	−0.03	−0.04	−0.05
Renal vascular disease, excluding vasculitis, *n* (%)	51 (7.5)	−0.02	−0.01	0.002
Diabetes mellitus, *n* (%)	34 (5.0)	0.02	−0.03	−0.02
Other multisystem disease, *n* (%)	32 (4.7)	0.01	0.01	0.02
Other, *n* (%)	18 (2.6)	−0.01	0.02	0.01
Unknown, *n* (%)	107 (15.7)	−0.01	0.01	0.01
Kidney transplant				
Pre-emptive transplantation, *n* (%)	105 (15.5)	0.03	0.08	0.06
Time between transplantation and baseline measurement, years	5.4 (1.9–12.0)	0.08 *	0.02	0.01
Male donor, *n* (%)	343 (50.4)	−0.07	−0.06	0.04
Donor age, years	46.0 (32.0–54.0)	−0.09 *	−0.04	0.00
Postmortal donor, *n* (%)	446 (65.6)	−0.03	0.07	0.08 *
Immunosuppressive therapy				
Prednisolone, %	673 (99.0)	−0.01	0.01	0.02
Prednisolone dose, mg	10.0 (7.5–10.0)	−0.04	−0.001	0.02
Tacrolimus, %	121 (17.8)	0.00	0.02	0.01
Cyclosporine, *n* (%)	269 (39.6)	−0.12 **	−0.12 **	−0.09 *
Azathioprine, *n* (%)	120 (17.6)	0.07	0.00	0.00
Mycophenolic acid, *n* (%)	446 (65.6)	−0.02	0.05	0.04
Everolimus/Sirolimus	13 (1.9)	−0.05	−0.02	0.01
Clinical variables				
Systolic blood pressure, mmHg	136 ± 17	−0.08 *	−0.08 *	−0.07
Diastolic blood pressure, mmHg	83 ± 11	−0.07	−0.01	−0.003
Heart rate, beats per minute	69 ± 12	−0.06	−0.06	−0.11 **
Antihypertensives, *n* (%)	598 (87.9)	−0.12 **	−0.12 **	−0.09 *
Renal function parameters				
Creatinine, umol/L	156 (112–181)	−0.13 ***	−0.12 **	−0.08 *
Cystatin C, mg/L	1.7 (1.3–2.2)	−0.17 ***	−0.23 ***	−0.20 ***
eGFR, mL/min/1.73 m^2^	41.0 ± 18.6	0.07	0.12 **	0.09 *
Proteinuria 0.5 g/day, *n* (%)	152 (22.4)	−0.05	−0.06	−0.07
Glucose homeostasis				
Glucose, mmol/L	5.3 (4.9–6.0)	−0.10 **	−0.13 ***	−0.15 ***
HbA1C, %	5.8 (5.5–6.2)	−0.07	−0.12 **	−0.14 ***
Diabetes mellitus, *n* (%)	162 (23.8)	−0.12 **	−0.15 ***	−0.18 ***
Antidiabetic medication, *n* (%)	105 (15.4)	−0.09 *	−0.09 *	−0.12 **
Serum parameters				
Albumin, g/L	43.0 ± 3.0	0.04	0.13 ***	0.16 ***
hs-CRP, mg/L	1.6 (0.7–4.6)	−0.04	−0.03	−0.09 *
Procalcitonin, ug/L	0.06 ± 0.06	−0.11 **	−0.15 ***	−0.12 **
Lipids				
Total cholesterol, mmol/L	5.1 ± 1.1	−0.09 *	0.04	0.06
LDL cholesterol, mmol/L	3.0 ± 0.9	−0.05	0.14 ***	0.15 ***
HDL cholesterol, mmol/L	1.3 (1.1–1.6)	0.35 ***	0.34 ***	0.37 ***
Triglycerides, mmol/L	1.7 (1.3–2.3)	−0.58 ***	−0.60 ***	−0.59 ***
Statin use, *n* (%)	359 (52.8)	0.05	−0.13 ***	−0.10 *
Liver parameters				
Total bilirubin, umol/L	10 (7–13)	0.03	0.07	0.12 **
ASAT, U/L	22 (18–27)	0.09 *	0.03	0.04
ALAT, U/L	19 (14–25)	0.001	−0.05	−0.04
Total protein, g/L	71.23 ± 5.1	−0.03	0.02	0.04
Gamma-GT, U/L	26 (19–41)	−0.03	−0.09 *	−0.11 **
Healthy lifestyle				
Current smoker, *n* (%)	81 (11.9)	−0.12 **	−0.08 *	−0.05
Alcohol intake, g/day	2.6 (0.0–11.1)	−0.07	−0.03	0.07
Physical activity, intensity x hours	5590 (3060–8415)	−0.01	0.07	0.09 *
Total energy intake, kJ/day	8713 (7172–10607)	−0.09	0.05	0.10 *
Metabolic syndrome (yes), *n* (%)	436 (64.1)	−0.19 ***	−0.28 ***	−0.31 ***
Fatty liver index, arbitrary units	56 (31–80)	−0.24 ***	−0.26 ***	0.29 ***
Dietary intake				
Peanuts, g/day	0.6 (0.0–3.4)	0.04	0.19 ***	0.23 ***
Peanut butter, g/day	0.0 (0.0–4.0)	0.01	0.13 **	0.13 **
Tree nuts, g/day	0.0 (0.0–3.9)	0.13 ***	0.18 ***	0.19 ***

Data are presented as mean ± SD, median (25th–75th percentile) or number (%). Univariate linear regression analyses were performed to obtain standardized βs and *p*-values for potential associations between baseline characteristics and circulating VLSFA. Abbreviations: BMI, body mass index; C20:0, arachidic acid; C22:0, behenic acid; C24:0, lignoceric acid; eGFR, estimated glomerular filtration rate; HbA1C, hemoglobin A1C; HDL, high-density lipoprotein; hs-CRP, high-sensitivity C-reactive protein; kJ, kilojoule; KTR, kidney transplant recipients; LDL, low-density lipoprotein; VLSFA, very long chain saturated fatty acids; * *p <* 0.05; ** *p* < 0.01; *** *p* < 0.001.

**Table 3:** Prospective analysis of standardized circulating VLSFA with all-cause mortality in KTR.

Models	C20:0, per 1-SD Relative Increment		C22:0, per 1-SD Relative Increment		C24:0, per 1-SD Relative Increment	
	HR (95% CI)	*p*	HR (95% CI)	*p*	HR (95% CI)	*p*
Model 1	0.78 (0.66–0.93)	0.001	0.65 (0.54–0.79)	<0.001	0.65 (0.54–0.79)	<0.001
Model 2	0.79 (0.67–0.95)	0.01	0.69 (0.57–0.84)	<0.001	0.68 (0.56–0.82)	<0.001
Model 3	0.80 (0.67–0.95)	0.01	0.71 (0.59–0.85)	<0.001	0.71 (0.59–0.86)	<0.001
Model 4	0.78 (0.64–0.94)	0.01	0.73 (0.60–0.89)	0.002	0.75 (0.61–0.92)	0.01
Model 5	0.77 (0.62–0.96)	0.02	0.70 (0.55–0.90)	0.01	0.73 (0.57–0.93)	0.01
Model 6	0.77 (0.63–0.93)	0.01	0.72 (0.59–0.89)	0.003	0.73 (0.60–0.90)	0.003
Model 7	0.85 (0.69–1.05)	0.14	0.79 (0.64–0.99)	0.04	0.80 (0.64–1.01)	0.06

Multivariable-adjusted Cox proportional-hazards regression analyses between circulating VLSFA and risk of all-cause mortality (*n*_events_ = 146) in KTR. Model 1: Adjusted for age and sex. Model 2: Model 1 + estimated glomerular filtration rate, time between transplantation and baseline measurement, primary renal disease and proteinuria. Model 3: Model 2 + albumin and procalcitonin. Model 4: Model 3 + smoking status and alcohol intake. Model 5: Model 4 + triglycerides. Model 6: Model 4 + use of cyclosporine. Model 7: Model 4 + systolic blood pressure, HDL-cholesterol, body mass index and diabetes status. Abbreviations: C20:0, arachidic acid; C22:0, behenic acid; C24:0, lignoceric acid; KTR, kidney transplant recipients; *n*_events,_ number of events.

**Table 4:** Prospective analysis of standardized circulating VLSFA with infectious disease mortality in KTR.

Models	C20:0, per 1-SD Relative Increment		C22:0, per 1-SD Relative Increment		C24:0, per 1-SD Relative Increment	
	HR (95% CI)	*p*	HR (95% CI)	*p*	HR (95% CI)	*p*
Model 1	0.58 (0.41–0.82)	0.002	0.48 (0.33–0.69)	<0.001	0.51 (0.35–0.73)	<0.001
Model 2	0.57 (0.41–0.80)	0.001	0.50 (0.35–0.72)	<0.001	0.53 (0.37–0.75)	<0.001
Model 3	0.55 (0.38–0.77)	<0.001	0.51 (0.35–0.73)	<0.001	0.55 (0.39–0.78)	<0.001
Model 4	0.57 (0.39–0.82)	0.002	0.52 (0.35–0.77)	0.001	0.57 (0.39–0.83)	0.004
Model 5	0.51 (0.32–0.81)	0.004	0.43 (0.26–0.72)	0.001	0.50 (0.31–0.82)	0.005
Model 6	0.58 (0.40–0.84)	0.004	0.52 (0.35–0.78)	0.001	0.57 (0.38–0.83)	0.004
Model 7	0.53 (0.35–0.82)	0.004	0.48 (0.30–0.75)	0.001	0.51 (0.33–0.80)	0.003

Multivariable-adjusted Cox proportional-hazards regression analyses between circulating VLSFA and risk of infectious disease mortality (*n*_events_ = 41) in KTR. Model 1: Adjusted for age and sex. Model 2: Model 1+ estimated glomerular filtration rate, time between transplantation and baseline measurement, primary renal disease and proteinuria. Model 3: Model 2 + albumin and procalcitonin. Model 4: Model 3 + smoking status and alcohol intake. Model 5: Model 4 + triglycerides. Model 6: Model 4 + use of cyclosporine. Model 7: Model 4 + systolic blood pressure, HDL-cholesterol, body mass index and diabetes status. Abbreviations: C20:0, arachidic acid; C22:0, behenic acid; C24:0, lignoceric acid; KTR, kidney transplant recipients; *n*_events,_ number of events.

## Data Availability

The data presented in this study are available on request from the corresponding author. The data are not publicly available due to confidentiality agreements.

## Supplementary Materials

The following are available online at https://www.mdpi.com/article/10.3390/nu13103383/s1, Figure S1. Participant flow chart of included KTR; Table S1. Prospective analysis of standardized circulating VLSFA with cardiovascular disease mortality in KTR; Table S2. Prospective analysis of standardized circulating VLSFA with cancer mortality in KTR.
