# Supplementary material for: Low Circulating Concentrations of Very Long Chain Saturated Fatty Acids Are Associated with High Risk of Mortality in Kidney Transplant Recipients
Source: Nutrients. 2021 Sep 26;13(10):3383. doi: 10.3390/nu13103383 (PMC8540190; doi:10.3390/nu13103383)
Supplement: Supplementary file 1 [file nutrients-13-03383-s001.zip › nutrients-1374590-supplementary.pdf]

Low Circulating Concentrations of Very Long Chain Saturated Fatty Acids Are Associated with High Risk of Mortality in Kidney Transplant Recipients

Fabian A. Vogelpohl [1,*], António W. Gomes-Neto [1], Ingrid A. Martini [2], Camilo G. Sotomayor [1], Dion Groothof [1], Maryse C.J. Osté [1], Rebecca M. Heiner-Fokkema [2], Frits A.J. Muskiet [2], Stefan P. Berger [1], Gerjan Navis [1], Ido P. Kema [2] and Stephan J.L. Bakker [1]

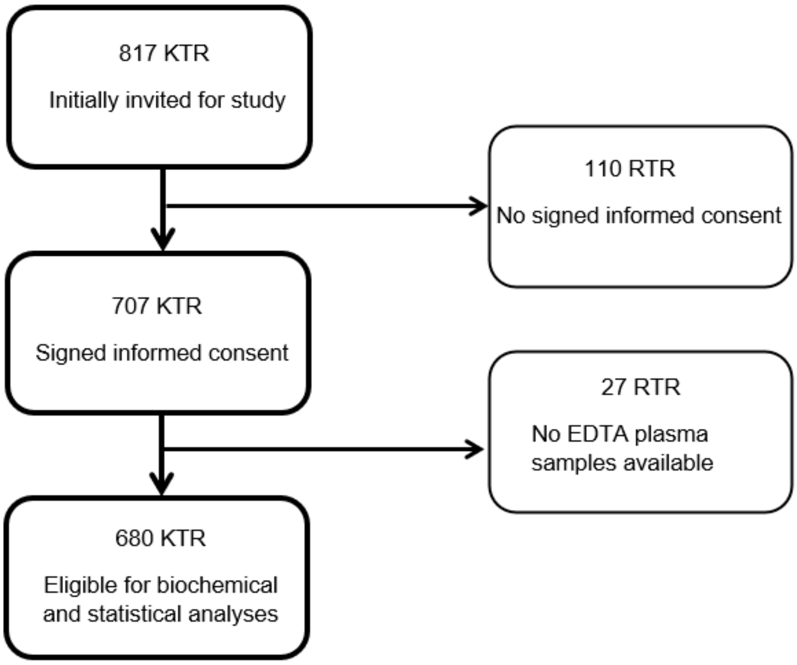

Figure S1. Participant flow chart of included KTR.

Table S1. Prospective analysis of standardized circulating VLSFA with cardiovascular disease mortality in KTR.

Models	C20:0, per 1-SD relative increment		C22:0, per 1-SD relative increment		C24:0, per 1-SD relative increment	
	HR (95% CI)	*p*	HR (95% CI)	*p*	HR (95% CI)	*p*
Model 1	1.02 (0.79-1.32)	0.89	0.77 (0.58-1.03)	0.08	0.68 (0.51-0.91)	0.009
Model 2	1.06 (0.81-1.37)	0.69	0.83 (0.62-1.11)	0.21	0.72 (0.53-0.97)	0.03
Model 3	1.06 (0.81-1.38)	0.69	0.84 (0.63-1.12)	0.23	0.73 (0.54-0.99)	0.04
Model 4	0.96 (0.72-1.28)	0.78	0.86 (0.63-1.17)	0.33	0.77 (0.56-1.06)	0.11
Model 5	1.04 (0.75-1.43)	0.84	0.90 (0.63-1.30)	0.58	0.78 (0.53-1.14)	0.20
Model 6	0.94 (0.70–1.36)	0.68	0.83 (0.61–1.13)	0.24	0.73 (0.53–1.01)	0.06
Model 7	1.18 (0.86-1.61)	0.30	1.05 (0.76-1.50)	0.76	0.96 (0.68-1.36)	0.82

Table S2. Prospective analysis of standardized circulating VLSFA with cancer mortality in KTR.

**Models**	**C20:0, per 1-SD relative increment**		**C22:0, per 1-SD relative increment**		**C24:0, per 1-SD relative increment**	
	**HR (95% CI)**	***p***	**HR (95% CI)**	***p***	**HR (95% CI)**	***p***
Model 1	0.73 (0.48-1.12)	0.15	0.80 (0.51-1.25)	0.33	0.87 (0.56-1.34)	0.52
Model 2	0.73 (0.47-1.13)	0.16	0.83 (0.52-1.32)	0.43	0.92 (0.58-1.44)	0.70
Model 3	0.76 (0.49-1.19)	0.23	0.87 (0.55-1.40)	0.57	1.00 (0.64-1.59)	0.98
Model 4	0.74 (0.46-1.19)	0.21	0.85 (0.52-1.38)	0.50	0.97 (0.61-1.56)	0.90
Model 5	0.67 (0.38-1.17)	0.16	0.79 (0.43-1.45)	0.45	0.97 (0.56-1.69)	0.91

Model 6	0.68 (0.41–1.13)	0.13	0.80 (0.48–1.34)	0.39	0.95 (0.58–1.57)	0.84
Model 7	0.72 (0.41-1.29)	0.27	0.92 (0.53-1.60)	0.76	1.10 (0.64-1.87)	0.73
